# Characterization and optimization of endogenous lipid accumulation in *Chlorella vulgaris* SDEC-3M ability to rapidly accumulate lipid for reversing nightly lipid loss

**DOI:** 10.1186/s13068-019-1493-9

**Published:** 2019-06-18

**Authors:** Feng Qi, Haiyan Pei, Ruimin Mu, Guixia Ma, Daoji Wu, Qiang Han

**Affiliations:** 1 School of Municipal and Environmental Engineering, Shandong Jianzhu University, Jinan, 250101 China; 2 Shandong Provincial Engineering Centre on Environmental Science and Technology, Jinan, 250061 China; 3 School of Environmental Science and Engineering, Shandong University, Qingdao, 266237 China; 4 Jinan Urban Construction Group Co., Ltd, Jinan, 250031 China; 5 Shandong Co-innovation Center of Green Building, Jinan, 250101 China

**Keywords:** Nightly lipid loss, Endogenous lipid accumulation, Microalgae, Light-NR/dark-ND process, Biodiesel

## Abstract

**Background:**

During inevitable light/dark cycle, lipid productivity of outdoor microalgae photoautotrophic cultivation is lowered by nightly biomass and lipid loss. To minimize, or even reverse the nightly lipid loss, it was expected that lipid accumulation would not cease, even if at night. Without relying on photosynthesis and organic matter in media, endogenous lipid accumulation that consumes energy and carbon sources derived from cells themselves, namely endogenous accumulation, is the only way for lipid production. The main aims of the present study was to characteristic endogenously accumulated lipid, confirm feasibility to reverse nightly lipid loss, and determine optimal conditions and its quality suitability for biodiesel feedstock production under stress conditions.

**Results:**

*Chlorella vulgaris* SDEC-3M ability to rapidly accumulated lipid under stress conditions was cultivated for 12 h in darkness, and the effects of various conditions on lipid accumulation and biomass loss were analyzed. Under non-stress conditions, lipid contents dropped. Under certain stress conditions, conversely, the lipid contents were substantially improved so that net nightly endogenous lipid accumulation was observed. Under the optimal conditions (aeration mode with 0.10 vvm and 15% CO_2_, 5–10 mg L^−1^ of NO_3_^−^-N, 30–35 °C, approximate 2500 mg L^−1^ of biomass), the lipid content was doubled and increased lipid was approximately 180 mg L^−1^. Among stress conditions, N-deficiency had the most significant effect on endogenous lipid accumulation, and the optimum results were characterized under relatively low-N concentrations. Higher consistency between loss in carbohydrate and gain in lipid confirmed accumulated lipid endogenously conversed from carbohydrate. Based on the analyses of fatty acids profiles and prediction of kinematic viscosity, specific gravity, cloud point, cetane number and iodine value, it was confirmed that the quality of lipid obtained under optimal conditions complied with biodiesel quality standards.

**Conclusion:**

Via triggering endogenous lipid accumulation by stress conditions, even in darkness, SDEC-3M can synthesize enough lipid suitable for biodiesel feedstock. It implies that the lipid accumulation phase in two-phase strategy can be scheduled at night, and following biomass production stage in light, which should be a solution to improve the lipid yield and quality of large-scale outdoor photoautotrophic microalgae cultivation for biodiesel production.

## Background

Concerns about limited fossil fuel reserves and climatic change have greatly aroused the interest of researchers for alternative energy sources. In this context, algae-based biodiesel becomes a focus due to its renewable and environment friendly properties [1–5]. Profiting from free carbon source from CO_2_ and free energy source for sunlight, little contamination, and its carbon neutrality, outdoor photoautotrophic cultivation has always been the main stream for microalgae biodiesel production [6–8]. However, light/dark cycle is an issue that must be considered in microalgal outdoor culture. Because photosynthesis is suspended and respiration keeps going at night, nightly biomass loss, and therefore, lipid loss become unavoidable [9–11]. Furthermore, the lipid accumulation was also delayed due to the imbalance of the metabolites related to lipid biosynthesis during light/dark cycle [7]. As a result, up to 35% loss in biomass [9] and 26% loss in triglyceride (TAG) [12] were observed in some microalgaes during darkness, which dramatically lowered the lipid productivity. For the purpose of low-cost algae-based biodiesel production, minimization and even reversal of nightly lipid loss in cells is necessary [13, 14]. Although a light-autotrophic/dark-heterotrophic cyclic cultivation was proposed to prevent nightly biomass from loss [10], it risks possible contamination, high cost of carbon source, and uncontrollable concentrations of organic substrates. Other researchers attempted to reduce the nightly biomass and lipid loss through optimizing cultivation conditions, such as controlling daytime and nighttime temperatures or avoiding mixing at night [9–11]. However, the attempts could not completely prevent any lipid loss.

Exposed to stress conditions, microalgae can be induced to accumulate lipid for biodiesel industry [5, 13–15]. The strategy has an associated problem that inhibits cell growth to decrease biomass [14]. The most feasible strategy to overcome this bottleneck is two-stage strategy, with a biomass production stage under suitable growth conditions, followed by a lipid accumulation stage under stress conditions [1, 16–19]. As the most common stress conditions used in the two-stage strategy [1, 20], N-deficiency caused by a medium excluding nitrogen or containing little nitrogen [12, 14, 19, 21] can induce cellular carbon flux is changed to lipid synthesis instead of carbohydrate and proteins [22], which drastically increases the lipid content in microalgae. It is hypothesized that induction by stress conditions accompanies with night, when the growth of microalgae is always ceased, can minimize, and even reverse the negative effect of nighttime on the lipid accumulation. Based on this hypothesis, a batch process comprising a light/dark cycle in cooperation with an N-rich/N-deficient cycle (light-NR/dark-ND) process modified two-stage strategy [19].

There are three key issues to be solved for a design of light-NR/dark-ND process. First, without relying on photosynthesis and organic matter in media, lipid synthesis in darkness consumes or reserves energy and carbon sources derived only from cells themselves, namely endogenous lipid accumulation. In the reported overwhelming studies, lipid content elevated by N-deficiency was observed in light, while the phenomenon was found in darkness only by a few researchers [7, 9, 20]. Thus, the feasibility of nightly endogenous lipid accumulation should be confirmed. Second, for most of the microalgae species, the obvious effects of lipid accumulation were usually observed within 2–6 days [21, 23]. For coordinating with light/dark cycle, however, noticeable endogenous lipid accumulation should occur just during about 12-h darkness. Fortunately, it was found that the lipid content of *Chlorella* was rapidly elevated only during 12 h N-deficiency, merely in light [19, 20]. Such rapid elevation of lipid content in microalgae cells occuring during darkness should be determined. The lipid accumulation can be improved by optimizing several cultivation conditions, such as temperature, light intensity, nutrient concentration and so on [5]. Thus, optimal conditions should also be determined. Carbon and energy sources of accumulated lipid should also be examined. Finally, Shekh et al. [18] found that stress-induced lipids were not suitable as biodiesel feedstocks, but some contrary findings were subsequently reported [16, 19, 24]. Thus, to confirm the suitability as feedstocks for biodiesel production, the quality of the endogenously accumulated lipid should be characterized via its fuel properties, such as kinematic viscosity (KV), specific gravity (SG), cloud point (CP), cetane number (CN), iodine value (IV). In view of the difficulty of direct measurements in smaller scale cultivation, the fuel properties were often indirectly determined. For example, based on the equations deduced by Hoekman et al. [21], these fuel properties can be predicted to be evaluated by fatty acid profiles that were relatively easy to measure [25, 26].

*Chlorella vulgaris* SDEC-3M is a UV mutant accumulating abundant carbohydrates (more than 40% of biomass) under N-rich conditions [27]. Its lipid content was almost doubled during 12 h N-deficiency, and the dominance of endogenous lipid accumulation has been proved by a comparison between increments of biomass and lipid [19]. The rapidly accumulating lipid suitable as biodiesel feedstocks was also confirmed [19]. However, the above results were all observed in light. Thus, as a potential model algae employed for light-NR/dark-ND process, its lipid yield and quality accumulated in darkness should be characterized.

In the present study, as a model microalgal strain, *C. vulgaris* SDEC-3M was cultivated for 12 h in darkness. Effects of the different conditions, such as blending mode, CO_2_ level, aeration rate, NO_3_^−^-N concentration, temperature and initial biomass concentration, lipid content, and lipid accumulation were characterized. The effect of different NO_3_^−^-N concentrations on carbohydrate content was also characterized. Based on these results, the feasibility of endogenous microalgal lipid accumulation in darkness and potential of lipid production under optimal cultivation conditions were confirmed, and its rough pathway was preliminary determined. Through fatty acid profile analysis, further, several properties of biodiesel derived from SDEC-3M were estimated to analyze their standard -compliance as biodiesel feedstocks. These findings provided new insight into design the light-NR/dark-ND process for enhancing microalgal lipid production during light/dark cycle, which was inevitable in outdoor cultivation.

## Methods

### Microalgae, culture medium, and aeration

As a potential CO_2_ biofixation and biofuel production candidate, *C. vulgaris* SDEC-3M was obtained from Shandong Provincial Engineering Centre on Environmental Science and Technology (SDEC) [27]. Seven modified SE media were used (denoted SE1 to SE7). Each 1 L of medium contained 75 mg of K_2_HPO_4_∙3H_2_O, 75 mg of MgSO_4_∙7H_2_O, 25 mg of CaCl_2_∙2H_2_O, 175 mg of KH_2_PO_4_, 25 mg of NaCl, 5 mg of FeCl_3_∙6H_2_O, 1 mL of A_5_ solution, 1 mL of Fe-EDTA, and 958 mL of deionized water. Each 1 L of A_5_ solution contained 2.86 g of H_3_BO_3_, 1.81 g of MnC1_2_∙4H_2_O, 0.22 g of ZnSO_4_∙4H_2_O, 79 mg of CuSO_4_∙5H_2_O, and 39 mg of (NH)_6_Mo_7_O_24_∙4H_2_O. Each 1 L of Fe-EDTA solution contained 10 g of Na_2_EDTA, 0.81 g of FeCl_3_∙6H_2_O, and 500 mL of 0.1 M HCl. Additionally, into each 1 L of SE2, SE4, SE5, SE6, and SE7 media were added 12.50, 31.25, 62.50, 125.00, and 250.00 mg NaNO_3_, respectively. Further, 40 mL of soil extract, which was supernatant filtered from boiled soil solution, was added to SE3, SE4, SE5, SE6, and SE7 media. The monitored values of NO_3_^−^-N concentration, and absence or presence of soil extract in the seven fresh media are shown in Table 1.

**Table 1:** NO_3_^−^-N concentrations and absence or presence of soil extract in the fresh media used in the present study

Medium	NO_3_^−^-N concentration (mg L^−1^)^a^	Soil extract^b^
SE1	0	−
SE2	2.06	−
SE3	2.00	+
SE4	6.52	+
SE5	11.43	+
SE6	21.13	+
SE7	42.81	+

^a^Monitoring data, NO_3_^−^-N from NaNO_3_ or nitrate in soil extract

^b^“−” represents media without soil extract, and “+” represents media with soil extract

Aeration was carried out by air or by mixtures of air and CO_2_ in different proportions that were prepared in industrial cylinders. The flow rates were adjusted by gas flow meters (Sevenstar, China).

### Pre-cultivation and re-suspension

Diluted to initial optical densities at wavelength 686 nm (OD_686_) of 0.35 cm^−1^ (biomass concentration of approximately 90 mg L^−1^) with fresh SE7 medium, SDEC-3M was inoculated in photobioreactors (ID, 120 mm; height, 300 mm; working volume, 2.5 L) [19, 28]. The photobioreactors were placed in a phytotron at 25 ± 1 °C under continuous 67.5 μmol m^−2^ s^−1^ illumination provided by six 40 W fluorescent daylight lamps on a panel. 15% CO_2_ (v/v) was bubbled into each photobioreactor through two 0.5 inch air stone diffusers at a flow rate of 0.008 vvm (volume gas per volume culture per minute). The microalgae with 11.56% of lipid content and 42.58% of carbohydrates content were harvested after 4 days’ pre-culturing. The cultures were centrifuged into microalgal pellets at 4000 rpm and −3 °C for 10 min. The microalgal pellets were washed to desalinate them and centrifuged into microalgal pellets again. Finally, the microalgal pellets washed were resuspended with the specific medium mentioned above, to regenerate culture suspension.

### Cultivation

Aside from a small portion retained as a control, most microalgae culture suspension regenerated was transferred into a 500 mL gas-washing bottle for “aeration” cultivation or sealed glass bottle with a lid for “standing” (i.e. quiescent) or “shaking” cultivation. These bottles were then covered with black cloth to protect from light in a constant temperature incubator for standing or aeration cultivation, or a shaking incubator for shaking cultivation at 130 rpm for 12 h in darkness.

To study the effect of blending mode, the microalgae were cultivated in standing mode without shake or aeration, shaking mode at 130 rpm, and aeration mode with 0.10 vvm of air, respectively, and all at 25 °C in SE3 medium. To study the effect of CO_2_ level, the microalgal broths were aerated with 0.10 vvm of air (approximately 0.04% CO_2_), and mixtures containing 1%, 5%, 15%, and 25% CO_2_, respectively, all at 25 °C in SE3 medium. To study the effect of aeration rate, the microalgae broths were aerated at 0, 0.04, 0.06, 0.10, 0.20 and 0.32 vvm with a mixture containing 15% CO_2_, respectively, likewise at 25 °C in SE3 medium. To study the effect of NO_3_^−^-N concentration and presence of soil extract in the medium, the microalgae were resuspended with the seven media, respectively, cultivated at 25 °C, and aerated at 0.10 vvm with a mixture containing 15% CO_2_. To study the effect of temperature, the microalgae were respectively cultivated at 10, 15, 20, 25, 30, 35 and 40 °C, in SE4 medium, and aerated at 0.10 vvm with a mixture containing 15% CO_2_. The above experiments were all low-density cultivation. To study the effect of biomass concentration, the microalgae were respectively cultivated with 701.2 (low-density, LD), 1461.5 (mid-density, MD), 2434.6 (high-density, HD) and 4869.2 (super-high-density, SD) mg L^−1^ of initial biomass concentration, in SE4 medium, at 30 °C, and aerated at 0.10 vvm with a mixture containing 15% CO_2_. The initial lipid contents were determined as approximately 11.7%. All treatments were triplicate.

### Measurement methodology

The retained microalgae culture suspensions were centrifuged to form microalgal pellets at 4000 rpm at − 3 °C for 10 min, as well as broths following 12 h darkness. Before centrifugation, the broths were sampled for cell observation using a microscope (CX31, Olympus, Japan), for determination of dissolved oxygen (DO) by an HQ-30D probe (Hach, USA) and pH by PHS-3C pH meter (Leici, shanghai, China). The pellets were then washed twice with distilled water to desalinate, dried, weighed, and ground into powder [24]. Biomass concentration (mg L^−1^) was obtained by comparing the dry biomass to the effective broth volume. Microalgal lipids were extracted by a chloroform/methanol (2:1, v/v) mixture, and their contents were estimated gravimetrically according to the previous report [24]. The total carbohydrate content of the microalgae was measured by the phenol–sulfuric acid method [29].

### Fatty acid profiles

The fatty acid profiles of the microalgae powder were analyzed by two steps including preparation of fatty acids methyl ester (FAME) and Gas Chromatography–Mass Spectrometry analysis (GC–MS) [24]. The samples were microalgae harvested before dark cultivation and after N-rich or N-deficient dark cultivation. Firstly, a one-step extraction transesterification method [30] with minor modification was employed to prepare FAME, which was carefully collected and analyzed with GC–MS (Trace GC-DSQII, Thermo Fisher). The compounds were identified by reference to the NIST Mass Spectral Database and quantified by the area normalization method.

### Important properties

The loss in the biomass concentration for 12 h in darkness (loss in biomass) was calculated in percentage via Eq. 1:

1 $${\text{Loss in biomass }}\left( \% \right) \, = \, \left( {X_{\text{I}} - X_{\text{F}} } \right) \, \times { 1}00 \, /X_{\text{I}}$$

where *X*_I_ (mg L^−1^) and *X*_F_ (mg L^−1^) are the initial and final biomass concentrations in the broths cultivated for 12 h in darkness, respectively.

The percentage of gain in lipid content for 12 h in darkness (gain in lipid content) was calculated via Eq. 2:

2 $${\text{Gain in lipid content }}\left( \% \right) \, = \, \left( {L_{\text{F}} - L_{\text{I}} } \right) \, \times { 1}00 \, /L_{\text{I}}$$

where *L*_I_ (%) and *L*_F_ (%) are initial and final lipid contents in cells cultivated for 12 h in darkness.

The percentage of loss in carbohydrate content for 12 h in darkness (loss in carbohydrate content) was calculated via Eq. 3:

3 $${\text{Loss in carbohydrate content }}\left( \% \right) \, = \, \left( {C_{\text{I}} - C_{\text{F}} } \right) \, \times { 1}00 \, /C_{\text{I}}$$

where *C*_I_ (%) and *C*_F_ (%) are initial and final carbohydrate contents in cells cultivated for 12 h in darkness.

The net lipid accumulation defined as variation of lipid concentrations for 12 h in darkness was calculated via Eq. 4:

4 $${\text{Lipid accumulation }}\left( {{\text{mg L}}^{ - 1} } \right) \, = X_{\text{F}} \times L_{\text{F}} - X_{\text{I}} \times L_{\text{I}}$$

where *X*_I_ × *L*_I_ (mg L^−1^) and *X*_F_ × *L*_F_ (mg L^−1^) are initial and final lipid concentrations in photobioreactor for 12 h in darkness.

The gain in lipid concentration defined as variation percentage of lipid concentrations during 12 h darkness was calculated via Eq. 5:

5 $${\text{Gain in lipid concentration }}\left( \% \right) \, = \, \left( {X_{\text{F}} \times L_{\text{F}} - X_{\text{I}} \times L_{\text{I}} } \right) \, \times { 1}00 \, /X_{\text{I}} \times L_{\text{I}}$$

Based on the equations deduced by Hoekman et al. [21], the biodiesel properties were predicted as follows:

Average degree of unsaturation (ADU) of microalgal oil was computed from fatty acid profiles via Eq. 6.

6 $${\text{ADU }} = \sum \, M \times Y_{i}$$

where *Y*_*i*_ is the mass fraction of each fatty acid constituent *i*, and *M* is the number of carbon–carbon double bonds in each fatty acid molecule.

The relationships between biodiesel ADU and other critical fuel properties, namely kinematic viscosity (KV, mm^2^ s^−1^ at 40 °C), specific gravity (SG, kg L^−1^), cloud point (CP, °C), cetane number (CN) and iodine value (IV, gI_2_/100 g), are shown in Eqs. 7–11 [24, 25]:

7 $$y_{ 1} = - 0. 6 3 1 6x + { 5}. 20 6 5\quad \quad R^{ 2} = \, 0. 6 70 4$$

8 $$y_{ 2} = \, 0.00 5 5x + \, 0. 8 7 2 6\quad \quad R^{ 2} = \, 0. 6 6 4 4$$

9 $$y_{ 3} = - 1 3. 3 5 6x + { 19}. 9 9 4\quad \quad R^{ 2} = \, 0. 6 80 9$$

10 $$y_{ 4} = - 6. 6 6 8 4x + { 62}. 8 7 6\quad \quad R^{ 2} = \, 0. 80 4 9$$

11 $$y_{ 5} = { 74}. 3 7 3x + { 12}. 7 1\quad \quad R^{ 2} = \, 0. 9 4 8 4$$

where *y*_1_, *y*_2_, *y*_3_, *y*_4_ and *y*_5_ are KV, SG, CP, CN and IV, respectively, and *x* is biodiesel ADU.

### Statistical analysis

The respective differences between losses in biomass, gains in lipid content, amounts of lipid accumulation, and fatty acid compositions were assessed using one-way analysis of variance (ANOVA). A difference was considered statistically significant when *p* < 0.05 according to Duncan’s tests.

## Results

### Effect of blending mode

Table 2 showed the percentage changes of the biomass concentration and lipid content, lipid accumulation in SDEC-3M, and dissolved oxygen in the broths in response to different blending modes during 12 h darkness. Both loss in biomass and gain in lipid content in SDEC-3M obtained in the aeration mode were much higher than those in other two modes. Further, because the gain in lipid content was much higher than the biomass loss, lipids were accumulated in the shaking and aeration modes, and the highest lipid accumulation of 31.20 ± 6.59 mg L^−1^ was obtained in the aeration mode.

**Table 2:** Loss in biomass, gain in lipid content and lipid accumulation in SDEC-3M, and dissolved oxygen in broths in response to different blending modes during 12 h darkness

Parameter	Blending mode		
	Standing	Shaking	Aeration
Loss in biomass (%)	0.64 ± 0.14^a^	1.64 ± 0.13^a^	7.14 ± 0.98^b^
Gain in lipid content (%)	0.57 ± 0.90^a^	8.17 ± 2.22^a^	48.25 ± 10.14^b^
Lipid accumulation (mg L^−1^)	−0.07 ± 0.62^a^	5.30 ± 1.79^a^	31.20 ± 6.59^b^
Dissolved oxygen (mg L^−1^)	1.67 ± 0.15	0.03 ± 0.06	6.83 ± 0.06

Each entry indicates the mean ± S.D., *n* = 3, measured from three independent cultures. Data in the same row followed by different letters are significantly different by Duncan’s test (*p* < 0.05)

Due to the closed cultivation, the standing mode meant no mixing and oxygenizing, while the shaking mode was supplied with mixing but no oxygenizing. Due to absence of mixing in the standing mode, most microalgae cells in the bottom of flasks precipitated and few suspended cells were observed in broths after 12 h darkness. As a result of no oxygenizing, almost zero DO was recorded in the broths for the shaking mode (Table 2). In aeration mode the two functions occurred simultaneously. The results show that mixing and oxygenizing, through which cells could receive nutrients and oxygen, are both necessary for cell activity and lipid accumulation, but significance analysis showed that the latter is much more important. In darkness, thus, aeration is essential to accumulate lipid in SDEC-3M.

### Effect of CO_2_ level

**Fig. 1:**
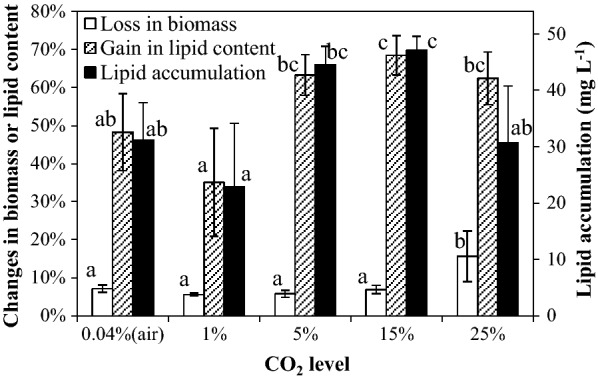
Loss in biomass, gain in lipid content and lipid accumulation in SDEC-3M in response to different CO_2_ levels during 12 h darkness. Each entry indicates the mean ± S.D., *n* = 3, measured from three independent cultures. Data for the same type of parameter (with the same shading) followed by different letters are significantly different by Duncan’s test (*p* < 0.05)

Figure 1 showed the percentage changed in the biomass concentration and lipid content, and lipid accumulation in SDEC-3M in response to different CO_2_ levels during 12 h darkness. No significant differences were observed between losses in biomass under 0.04% CO_2_ (air), 1% CO_2_, 5% CO_2_, or 15% CO_2_, while these were significantly lower than the losses occurring under 25% CO_2_. Some dead cell walls were observed in broths aerated with 25% CO_2_, but were scarce in other treatments. This indicated that the reason causing increase of biomass loss was not more active respiration but more death and autolysis of some microalgae cells. This could imply that the tolerance of SDEC-3M to CO_2_ above 15% was poorer.

The observation that higher lipid contents were obtained under higher CO_2_ level was consistent with the reported findings under continuous illumination or light/dark cycles [20]. It showed that a high CO_2_ level stimulated lipid accumulation in microalgae in both light and darkness. High CO_2_ level is considered a stress condition to promote lipid accumulation for some algae species [3, 31]. The other possible explanation is that lower O_2_ partial pressure in higher CO_2_ mixtures lowers the DO in the broths, which is lethal to algal cells [1] and eventually decreases the lipid content in microalgae cells [32].

No significant differences were found between gains in lipid content under 5%, 15%, and 25% CO_2_. It indicated that the effect of the CO_2_ level could be neglected when above 5%. Moreover, it was observed that gain in lipid content with air was higher than that under 1% CO_2_. A possible reason is that SDEC-3M is a high-CO_2_-requiring (HCR) mutant [27], for which low CO_2_ level (as in air) is a stress condition that also stimulates lipid accumulation in cells. Thus, high gain in lipid content and low loss in biomass coexisted under 5 to 15% CO_2_, which is optimal for lipid production (no significant difference). The highest lipid accumulation of 47.13 ± 2.47 mg L^−1^ was obtained with 15% CO_2_.

### Effect of aeration rate

**Fig. 2:**
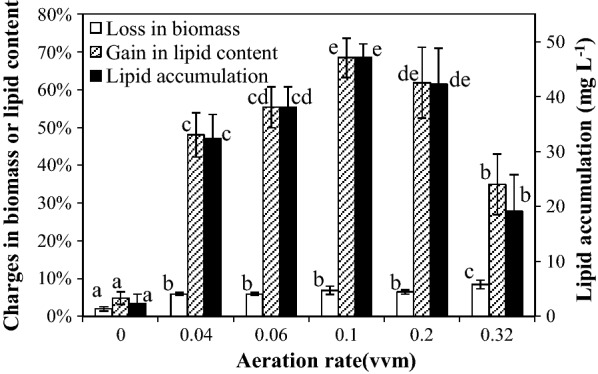
Loss in biomass, gain in lipid content and lipid accumulation in SDEC-3M in response to different aeration rates during 12 h darkness. Each entry indicates the mean ± S.D., *n* = 3, measured from three independent cultures. Data for the same type of parameter (with the same shading) followed by different letters are significantly different by Duncan’s test (*p* < 0.05)

Figure 2 showed the percentage changed in the biomass concentration and lipid content, and lipid accumulation in SDEC-3M in response to different aeration rates during 12 h darkness. No significant differences were observed between loss in biomass at different aeration rates, except at ‘no aeration’ and 0.32 vvm aeration. A possible reason is that the ‘no aeration’ condition inhibited cells’ respiratory activity with an absence of mixing and oxygenation (“Effect of blending mode” in section), while the higher aeration rate of 0.32 vvm might damage microalgal cells due to intense turbulence [28].

A moderate aeration rate of 0.10 vvm resulted in the maximum lipid content, implying that a moderate aeration rate favors lipid accumulation in darkness. Similar results were also observed in darkness [20] or in light [28, 33] in previous research. This could be considered as a reason that both inefficient mixing at low aeration rate that limits microalgal uptake of nutrients and the damage at high aeration rate to the cells by intense turbulence each inhibits the metabolism of lipid synthesis [28].

Because of the slight difference of loss in biomass at different aeration rates, lipid accumulation depended more on gain in lipid content. This result was also confirmed through the same results by performing ANOVA on gain in lipid content and lipid accumulation (Fig. 2). Therefore, the highest lipid accumulation of 47.13 ± 2.47 mg L^−1^ was obtained at 0.10 vvm, with significantly higher gain in lipid content. Generally, the moderate aeration rate of 0.10 vvm was the optimal condition.

### Effect of NO_3_^−^-N concentration and soil extract in media

**Fig. 3:**
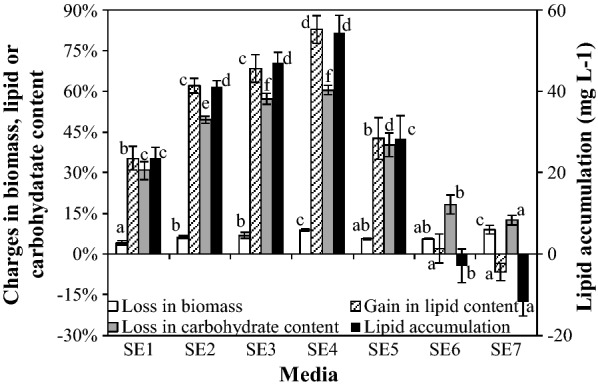
Loss in biomass, gain in lipid content, loss in carbohydrate content and lipid accumulation in SDEC-3M in response to different media during 12 h darkness. The NO_3_^−^-N concentrations and absence or presence of soil extract for each medium is given in Table 1. Each entry indicates the mean ± S.D., *n* = 3, measured from three independent cultures. Data for the same type of parameter (with the same shading) followed by different letters are significantly different by Duncan’s test (*p* < 0.05)

Figure 3 showed the percentage changed in the biomass concentration, lipid content and carbohydrate content, and lipid accumulation in SDEC-3M in response to different media during 12 h darkness. Loss in biomass fluctuated in the range of 4–8% in different media. It show that the short-term effect of NO_3_^−^-N concentrations and soil extract on biomass loss in darkness is limited.

Even in darkness, as Fig. 3 shows, the loss of lipid contents were reversed from − 6.66 ± 3.22% under N-deficiency to 82.86 ± 5.05% under N-rich condition, so that net nightly lipid accumulation was achieved. Significant higher gains in lipid contents and lipid accumulations were obtained under relatively low NO_3_^−^-N concentrations (in SE2, SE3, and SE4 medium), rather than under completely N-free conditions (in SE1 medium). Although it is well known that large amount of lipids can be accumulated in microalgae under N-deficiency [34], the relatively low nitrogen concentration could be more favorable to lipid accumulation in some microalgae species, which were reported by some previous researchers [20, 35, 36]. Our findings confirm that the conclusion is also valid for endogenous lipid accumulation by microalgae cultivated in darkness. These phenomena may be caused by the reason that nitrogen level should be low enough to trigger the metabolism towards lipid in cells [37], and not to suspending the synthesis of the vital enzymes related to lipid synthesis [19]. As the results showed, relatively low amounts of nitrogen, such as 5 to 10 mg L^−1^ NO_3_^−^-N, is optimal, and the highest gain in lipid content of 82.86 ± 5.05% and greatest lipid accumulation of 54.49 ± 4.21 mg L^−1^ were obtained in SE4 medium with 6.52 mg L^−1^ NO_3_^−^-N.

As Fig. 3 shows, carbohydrate contents in SDEC-3M in seven media declined in varying degrees. Interestingly, in media with more or less nitrogen, loss in carbohydrate content was significantly lessened, and gain in lipid content and lipid accumulation had the same trend, even though the former was not like the latter two showing negative values in N-rich media. The highest loss in carbohydrate content of 60.47 ± 1.74% was found in SE4, in which highest gain in lipid content and lipid accumulation were obtained.

Besides nitrogen, some other ingredients were added to the media with the soil extract. To investigate the possible interference of these ingredients, the results obtained in SE3 medium with added soil extract but without additional NaNO_3_ were compared with those for SE2 medium with equivalent NO_3_^−^-N. The attained lipid content of 68.47 ± 5.18% in SE3 medium was slightly higher than that of 62.14 ± 2.72% in SE2 medium, but there were no significant differences between them (*p* > 0.05). The same trend was observed between lipid accumulations in SE2 medium and SE3 medium. The results show that the positive effects of other ingredients in soil extract on lipid accumulation were not significant, compared with the effect of nitrogen.

### Effect of temperature

**Fig. 4:**
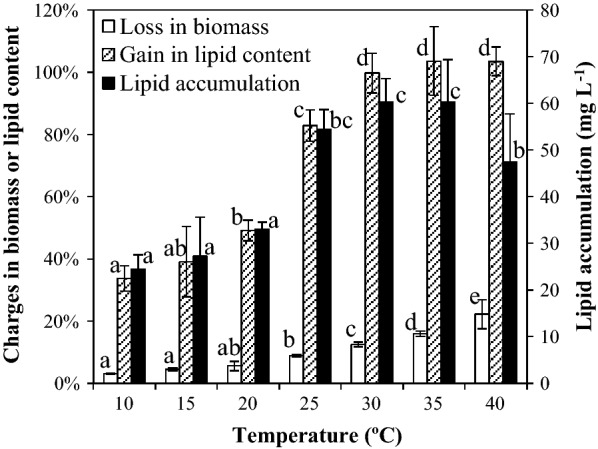
Loss in biomass, gain in lipid content and lipid accumulation in SDEC-3M in response to different temperatures during 12 h darkness. Each entry indicates the mean ± S.D., *n* = 3, measured from three independent cultures. Data for the same type of parameter (with the same shading) followed by different letters are significantly different by Duncan’s test (*p* < 0.05)

Figure 4 showed the percentage changed in the biomass concentration and lipid content, and lipid accumulation in SDEC-3M in response to different temperatures imposed while culturing for 12 h in darkness. The biomass loss increased as temperature increased, and the highest loss was 22.21 ± 4.71% at 40 °C, when a few dead cells walls were observed in the broths. It indicated that the SDEC-3M cells were more active at higher temperatures, but are not tolerant to temperatures beyond 40 °C.

Whether in low temperatures range (10–20 °C) or high temperatures range (25–40 °C), gain in lipid content slightly rose with increasing temperatures, while between those two ranges it dramatically increased from 49.14 ± 3.37% (20 °C) to 85.26 ± 5.11% (25 °C), which yields a statistically significant difference. In 25 to 40 °C, the lipid contents doubled. Thus, the highest lipid accumulation of 60.32 ± 5.03 and 60.25 ± 9.14 mg L^−1^ was obtained at 30 and 35 °C (no significant difference), even though losses in biomass were up to 12.48 ± 0.73% and 15.94 ± 0.82%. Due to higher loss in biomass, lipid accumulation was lower at the highest temperature (40 °C).

Previous studies confirmed that the effect of nutrient limitation on lipid content in cells is more significant than the effect of temperature stress [2, 15]. In the present study, although gains in lipid content were improved from 33.76 ± 4.06% to 103.59 ± 11.02% through adjusting temperature, by comparison, as the more dominant contribution, N- deficiency reversed the loss of lipid contents under N-rich condition (from − 6.66 ± 3.22% to 82.86 ± 5.05%). However, it is important to find a wide temperature range suitable for lipid production, after all it is difficult to control temperature in outdoor cultivation [1, 38]. The evidence that the lipid contents in SDEC-3M were greatly increased across a wide range of ‘high’ temperatures suggests it is an outstanding candidate for lipid production.

### Effect of biomass concentration

**Fig. 5:**
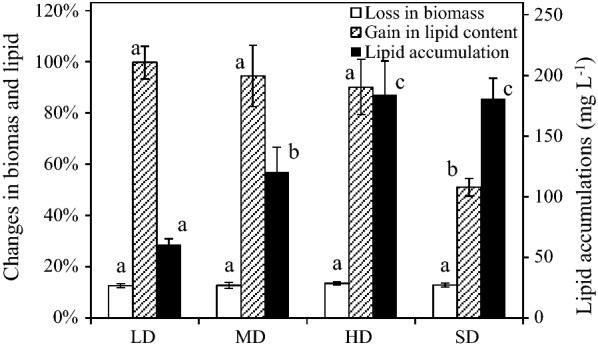
Loss in biomass, gain in lipid content and lipid accumulation in SDEC-3M in low, medium, high and super-high-density cultivation (presented as LD, MD, HD and SD) during 12 h darkness. Each entry indicates the mean ± S.D., *n* = 3, measured from three independent cultures. Data for the same type of parameter (with the same shading) followed by s are significantly different by Duncan’s test (*p* < 0.05)

Figure 5 showed the percentage changed in the biomass concentration and lipid content, and lipid accumulation in SDEC-3M in response to initial biomass concentration for 12 h in darkness. No significant differences of losses in biomass were observed between in different density cultivation. This indicated that the proportion of biomass loss was scarcely affected by the cultivation density.

As Fig. 5 shows, gain in lipid content significantly declined when biomass concentration was over 2500 mg L^−1^ (SD). Based on higher initial biomass concentration, however, the higher lipid accumulations of approximate 180 mg L^−1^ were both obtained in HD and SD cultivation, without significant differences between them. The lipid accumulations in SD and HD were obtained by 13.40% or 12.79% of loss in biomass and 90.14% or 50.97% of gain in lipid content, respectively. Therefore, in terms of gain in lipid concentration. 31.66 ± 2.98% in SD was considerably lower than 64.66 ± 9.64% in HD. Obviously, higher cultivation density can increase lipid production efficiency of bioreactor, but will be counteracted by decline of lipid content when the cultivation density was higher than 2500 mg L^−1^. Microalgae biomass concentrations generally remain at 2 to 8 g L^−1^ in closed photoautotrophic systems [6]. Thus, the optimal biomass concentration (approximately 2500 mg L^−1^) is maintained by promoting microalgal growth rate in daytime and optimizing medium renewal rate is a key in outdoor cultivation.

### Fatty acid profiles

Table 3 showed the fatty acid profiles of lipid in SDEC-3M before and after N-rich (SE7 medium) and N- deficient (in SE4 medium) 12 h dark cultivation. C16–C18 fatty acids (16:0, 16:1, 16:2, 18:0, 18:1, 18:2 and 18:3) suitable for biodiesel production [24, 39] were all higher than 95% and 16:0 was the most prevalent constituent in the three samples, followed by 18:1 and 18:2. ANOVA showed that 18:1 underwent statistically significantly increased, 16:0 and 18:3 underwent statistically significant decreased, and 18:2 and non-C16/C18 fatty acids (other) exhibited no statistically significant change under both conditions, while 16:1 and 18:0 changed more during N-deficient cultivation than they did during N-rich cultivation. The results that the significant effect of nutrient limitation on fatty acid profiles were also found in some previous studies [18, 22, 40].

**Table 3:** Fatty acid profiles of lipids derived from SDEC-3M samples before and after 12 h N-rich or N-deficient cultivation in darkness

Lipid number	Relative prevalence		
	Before cultivation	After N-rich cultivation	After N-deficient cultivation
16:0	47.84 ± 1.52%^b^	43.23 ± 1.47%^a^	43.20 ± 1.21%^a^
16:1	1.80 ± 0.88%^b^	0.54 ± 0.35%^a^	0.95 ± 0.41%^ab^
16:2	3.62 ± 0.66%^b^	4.97 ± 0.30%^c^	2.57 ± 0.55%^a^
18:0	1.16 ± 1.16%^a^	1.24 ± 1.24%^a^	2.48 ± 2.48%^b^
18:1	8.11 ± 1.35%^a^	18.75 ± 2.67%^b^	20.02 ± 1.04%^b^
18:2	19.59 ± 1.43%^a^	19.08 ± 2.78%^a^	18.22 ± 0.66%^a^
18:3	13.72 ± 0.87%^b^	8.78 ± 0.18%^a^	8.67 ± 0.92%^a^
Other	4.17 ± 0.4%^a^	3.41 ± 0.5%^a^	3.89 ± 0.6%^a^

Each entry indicates the mean ± S.D., *n* = 3, measured from three independent cultures. Data in the same row followed by different letters are significantly different by Duncan’s test (*p* < 0.05)

## Discussion

### Feasibility of nightly endogenous lipid accumulation

Under the photoautotrophic mode, the management of the stress conditions, especially N-deficiency are common strategies for improving lipid accumulation [5, 13, 16, 20, 41]. In the present study, similar results occurring at night confirm the feasibility of nightly endogenous lipid accumulation. Due to no carbon source and energy source supplied by photosynthesis or organic matter in media, triggering endogenous lipid accumulation should be the only one way to minimize, even reverse nightly lipid loss caused by light/dark cycle. In consideration of growth ceasing in darkness, growth inhibition caused by stress conditions is disappeared at night. Based on these, the light-NR/dark-ND process can eliminates the negative effects of nighttime on lipid production, and then increases lipid productivity.

### Pathway of nightly endogenous lipid accumulation

It is observed that carbon and energy fixed by photosynthesis are shifted from starch synthesis to lipid synthesis under stress conditions, which pathway and mechanism are largely clearer [33, 37, 42, 43]. Relative to photosynthetic lipid accumulation, few nightly endogenous lipid accumulation in microalgae cells have been researched. The dramatic improvement of lipid content [20] and a conversion from sugar to lipids [41] in *Chlorella* were observed under N-deficiency and darkness. In present study, it is found that loss in carbohydrate content had the same trend as gain in lipid content in media with different nitrogen concentrations, and their maximum value were both obtained under relatively low nitrogen concentrations. Considering that the nightly respiration of microalgae cells is mainly supported by starch, rather than lipid [44] and abundant carbohydrates in SDEC-3M accumulating under N-rich conditions (more than 40% of biomass), the results confirm that the energy sources and carbon sources used for lipid accumulation are from carbohydrates. However, the detailed knowledge of endogenous pathway from carbohydrates to lipid is limited, which forces us to test microalgal strains one by one. Hence, it is necessary to clarify the pathway and mechanism of nightly endogenous lipid accumulation.

### Optimal conditions and maximum yield of nightly endogenous lipid accumulation

After one condition after another compared, the optimal condition for more lipid accumulation are followed: aeration mode, moderate aeration rate (0.10 vvm), higher CO_2_ level (15%), relatively low NO_3_^−^-N concentrations (5–10 mg L^−1^), higher temperature (30–35 °C) and higher density cultivation (approximate 2500 mg L^−1^). These results do not show that more unfavorable conditions are, the more lipid microalgal can be accumulated. The reasons should be that too severe conditions lead to cell death and suspension of lipid synthesis. Under optimal conditions, over 180 mg L^−1^ was accumulated during 12 h darkness, with 13.40% of loss in biomass, 90.14% of gain in lipid content and 64.66% of gain in lipid concentration. The result exceed lipid productivities of the most promising oleaginous microalgae under photoautotrophic culturing [2, 16, 28, 38, 40, 45], although nightly biomass loss is still inevitable. The result shows the great potential of SDEC-3M and light-NR/dark-ND batch process in application for biodiesel production.

### Biodiesel performance derived from nightly endogenous lipid

Whether lipid in SDEC-3M are commercial feedstocks for biodiesel production depends on not only lipid yield, but also lipid quality. In many studies, the biodiesel performances were analyzed by properties derived by fatty acid profiles [18, 19, 24, 25]. According to Eqs. 7 to 11, biodiesel ADU and five other critical fuel properties (KV, SG, CP, CN and IV), can be directly or indirectly calculated from fatty acid profiles. As shown in Table 4, due to the change in fatty acid profiles, AUD, SG and IV decreased, whereas KV, CP, and CN rose during 12 h dark cultivation. All changes were more significant under N-deficient conditions. Because CN, IV, and CP relate to combustion performance, oxidation stability, and low temperature performance of biodiesel, respectively, while KV and SG affect emission levels and combustion performance [4, 19, 24, 39], the combustion performance, oxidation stability, and exhaust emission levels of biodiesel would be improved, but the low temperature performance would worsen following 12 h dark cultivation, which would be more obvious under N-deficient conditions. Despite those changes, the biodiesel properties of all three samples still complied with the three most common biodiesel quality standards, including GB/T 20828 in China, ASTM D6751 in the USA, and EN 14214 in Europe (Table 4). The results indicate that endogenously accumulated lipid in darkness by SDEC-3M as biodiesel feedstocks are feasibility.

**Table 4:** Six fuel properties of biodiesel—ADU, KV, SG, CP, CN and IV—derived from SDEC-3M samples before and after 12 h N-deficient or N-rich cultivation in darkness, and their standards in China, the USA, and Europe

Biodiesel property	Empirical estimation			Standard value [19]		
	Before cultivation	After N-rich cultivation	After N-deficient cultivation	CN (GB/T 20828)	USA (ASTM D6751)	Europe (EN 14214)
KV 40 °C (mm^2^ s^−1^)	4.56 ± 0.02	4.59 ± 0.02	4.62 ± 0.02	4.39 to 4.95	1.9 to 6.0	1.9 to 6.0
SG (kg L^−1^)	0.8782 ± 0.0002	0.8779 ± 0.0002	0.8777 ± 0.0002	0.875 to 0.880	0.82 to 0.90	0.87 to 0.89
CP (°C)	6.41 ± 0.47	7.03 ± 0.49	7.69 ± 0.41	2.83 to 14.66	Report	Report
CN	56.09 ± 0.23	56.4 ± 0.25	56.73 ± 0.21	54.31 to 59.29	min 49	min 47
IV (gI_2_/100 g)	88.37 ± 2.6	84.9 ± 2.75	81.24 ± 2.29	42.41 to 108.27	–	–
ADU	1.02 ± 0.03	0.97 ± 0.04	0.92 ± 0.03	0.40 to 1.28	–	–

Each entry indicates the mean ± S.D., *n* = 3, which was measured from three independent cultures

## Conclusions

In the present study, *C. vulgaris* SDEC-3M was cultivated for 12 h in darkness. Under non-stress conditions, lipid contents dropped, which caused more than 10 mg L^−1^ of lipid loss, together with biomass loss. However, endogenous lipid accumulation triggered by stress conditions drastically increased lipid content. Based on the lipid content gain overwhelming the biomass loss, lipid loss was minimized, and even reversed. Under optimal conditions (aeration mode, moderate aeration rate, higher CO_2_ level, relatively low NO^3−^-N concentrations, higher temperature and higher density cultivation), the lipid content was elevated by 90.14% and proximately 180 mg L^−1^ net accumulated lipid (approximately 65% increment based on initial lipid concentration) was obtained. The estimated properties complied with biodiesel standards. It indicated that lipids from SDEC-3M were suitable feedstocks for biodiesels. These findings determining the feasibility and optimal conditions of endogenous lipid accumulation in SDEC-3M in darkness, are the preliminary effort to commercialize the light-NR/dark-ND process for microalgal biodiesel production.

## Data Availability

All data generated or analyzed during the present study are included in this published article.

## Abbreviations

Light-NR/dark-ND process: a process combining a light/dark cycle in cooperation with an N-rich/N-deficient cycle
SDEC: Shandong Provincial Engineering Centre on Environmental Science and Technology
UV: ultraviolet
DO: dissolved oxygen
LD: low-density
MD: mid-density
HD: high-density
SD: super-high density
FAME: fatty acids methyl ester
GC-MS: Gas Chromatography–Mass Spectrometry analysis
ADU: average degree of unsaturation
KV: kinematic viscosity
SG: specific gravity
CP: cloud point
CN: cetane number
IV: iodine value
Eqs: equations
ANOVA: one-way analysis of variance
S.D.: standard deviation
